# Linking minimum inhibitory concentrations to whole genome sequence-predicted drug resistance in *Mycobacterium tuberculosis* strains from Romania

**DOI:** 10.1038/s41598-018-27962-5

**Published:** 2018-06-26

**Authors:** Carolien Ruesen, Anca Lelia Riza, Adriana Florescu, Lidya Chaidir, Cornelia Editoiu, Nicole Aalders, Dragos Nicolosu, Victor Grecu, Mihai Ioana, Reinout van Crevel, Jakko van Ingen

**Affiliations:** 1 Department of Internal Medicine, Radboud university medical center, Nijmegen, The Netherlands; 2 Human Genomics Laboratory, University of Medicine and Pharmacy of Craiova, Craiova, Romania; 3 “Victor Babes” Infectious Diseases and Pneumophtisiology Hospital Craiova, Dolj County, Romania; 4 Health Research Unit, Faculty of Medicine, Padjadjaran University/Hasan Sadikin Hospital, Bandung, Indonesia; 5 Department of Medical Microbiology, Radboud university medical center, Nijmegen, The Netherlands

## Abstract

*Mycobacterium tuberculosis* drug resistance poses a major threat to tuberculosis control. Current phenotypic tests for drug susceptibility are time-consuming, technically complex, and expensive. Whole genome sequencing is a promising alternative, though the impact of different drug resistance mutations on the minimum inhibitory concentration (MIC) remains to be investigated. We examined the genomes of 72 phenotypically drug-resistant *Mycobacterium tuberculosis* isolates from 72 Romanian patients for drug resistance mutations. MICs for first- and second-line drugs were determined using the MycoTB microdilution method. These MICs were compared to macrodilution critical concentration testing by the Mycobacterium Growth Indicator Tube (MGIT) platform and correlated to drug resistance mutations. Sixty-three (87.5%) isolates harboured drug resistance mutations; 48 (66.7%) were genotypically multidrug-resistant. Different drug resistance mutations were associated with different MIC ranges; *katG* S315T for isoniazid, and *rpoB* S450L for rifampicin were associated with high MICs. However, several mutations such as in *rpoB*, *rrs* and *rpsL*, or *embB* were associated with MIC ranges including the critical concentration for rifampicin, aminoglycosides or ethambutol, respectively. Different resistance mutations lead to distinct MICs, some of which may still be overcome by increased dosing. Whole genome sequencing can aid in the timely diagnosis of *Mycobacterium tuberculosis* drug resistance and guide clinical decision-making.

## Introduction

Drug-resistance poses a major threat to tuberculosis (TB) control. Global surveillance data suggest that in 2015, there were an estimated 480.000 new cases of multidrug-resistant TB (MDR-TB), i.e. resistant to at least isoniazid (INH) and rifampicin (RIF), and that 9.5% of these cases were extensively drug-resistant (XDR), i.e. also resistant to amikacin (AMI), kanamycin (KAN), or capreomycin (CAP), and at least one fluoroquinolone (FLQ)^1^. It is estimated that less than half of these cases are detected, and an even smaller proportion receive appropriate treatment^1^. One of the main problems in the control of drug-resistant TB (DR-TB) is the lack of laboratory capacity to diagnose resistance^2^. Conventional culture-based drug susceptibility testing (DST) is expensive, time-consuming and requires a specialized biosafety laboratory. Methods for DST for many of the second-line drugs have not yet been fully standardized^3^. In addition, these phenotypic methods often rely on growth of mycobacterial cultures in the presence of a single ‘critical’ drug concentration to distinguish resistant and susceptible strains based on epidemiological breakpoints, which are near the wild type minimum inhibitory concentration (MIC) distribution for some drugs^4^.

Molecular assays are now available and can overcome some of the disadvantages of phenotypic methods to diagnose DR-TB. Yet, these assays are expensive and cannot identify all of the genetic loci associated with drug resistance^5,6^. Whole genome sequencing (WGS) of *M*. *tuberculosis*, tackles this problem and could enable the reliable prediction of the drug susceptibility phenotype within a clinically relevant timeframe. A fundamental issue in the application of WGS to predict drug resistance is how to interpret mutations in relation to phenotypic antibiotic resistance. The level of resistance, reflected by the MIC, a single nucleotide polymorphism (SNP) causes is most important for clinicians treating patients, in order to determine whether to increase the dosage or change the regimen.

Therefore, in the current study we provide this missing link by comparing MICs to drug resistance mutations determined by WGS. For this purpose we used a collection of drug-resistant strains from Romania, which had 20,000 new TB cases, and 800 new MDR-TB cases in 2012, making it one of the 18 high-priority countries for TB control in the World Health Organization (WHO) European Region^7^.

## Methods

### Selection of *M. tuberculosis* isolates

We used *M*. *tuberculosis* isolates from the bio-archive established at the TB laboratory of the Clinical Hospital of Infectious Diseases and Pneumophtisiology “Victor Babes” Craiova between 2014 and 2016. The laboratory runs approximately 7,000 sputum samples per year, covering much of the TB epidemic in Dolj county. Routine diagnostics as indicated by the National Control Programme include sputum microscopy and culture on Löwenstein-Jensen medium (liquid media introduced in May 2015). After primary culture on Löwenstein-Jensen medium, screening for INH and RIF-resistance is performed using INH 0.2 mg/l and RIF 40 mg/l-supplemented Löwenstein-Jensen slants.

All 73 isolates that were phenotypically resistant to INH and/or RIF were selected for the purpose of the present study. All 73 isolates were from different patients, and no serial isolates were included. Isolates stored in trypticase soy broth supplemented with glycerol were sub-cultured on Löwenstein-Jensen medium. Sub-cultured colonies were used for DNA extraction, and susceptibility testing.

### Phenotypic drug susceptibility testing

All isolates were simultaneously subjected to MIC determinations of rifampicin, rifabutin, ethambutol, streptomycin, amikacin, kanamycin, ofloxacin, moxifloxacin, para-aminosalicylic acid (PAS), ethionamide and cycloserine by broth microdilution in Middlebrook 7H9 broth (SensiTitre MycoTB assay; Trek Diagnostics/ThermoFisher, Landsmeer, the Netherlands). Every isolate was tested once, unless contamination or no growth was observed; then the isolate was re-cultured and once more subjected to MIC testing. The MycoTB assay is a new method for susceptibility testing of *M*. *tuberculosis* complex^8,9^. The plate uses a 96-well microtiter broth format and contains 12 lyophilized first- and second-line anti-mycobacterial drugs. In contrast to other *M*. *tuberculosis* complex susceptibility methods, which test one or two critical concentrations of a drug, the MycoTB assay examines a range of drug concentrations and produces an MIC result. In addition, critical concentration testing for streptomycin, isoniazid, rifampicin and ethambutol (SIRE assay; BD BioScience, Erembodegem, Belgium) was performed using the Mycobacterium Growth Indicator Tube (MGIT) macrodilution platform (BD BioScience, Erembodegem, Belgium). The critical concentrations used for MGIT testing were 0.10 µg/ml for INH, 1.00 µg/ml for RIF, 5.00 µg/ml for EMB, and 1.00 µg/ml for STR. Technicians performing susceptibility tests were blinded to the sequencing results.

### Whole genome sequencing and *in silico* determination of drug resistance

DNA isolation using UltraClean® Microbial DNA Isolation Kit (MO BIO Laboratories Inc., Carlsbad, CA) was followed by quantitative (Qubit v3.0) and qualitative assessment of the DNA (gel electrophoresis). We used the Nextera XT DNA Library Preparation Kit (Illumina, San Diego, CA) for library preparation. *M*. *tuberculosis* DNA was sequenced on an Illumina NextSeq500 instrument using 2 × 150 bp paired-end reads. One isolate was excluded due to failed library preparation, resulting in a total set of 72 isolates. No internal control was performed for WGS. After sequencing, the raw FASTQ sequence reads were filtered using Trim Galore (http://www.bioinformatics.babraham.ac.uk/projects/trim_galore), including removing of adapter sequences. Sequencing coverage was determined using the FASTQC quality control tool version 0.10.1, and the Genome Analysis Toolkit^10^. The average proportion of bases sequenced with a sequencing error rate of 0.1% or less per base was 77.8% per genome. The average coverage depth for the 72 sequenced strains was 165.1, and the average percentage of bases covered by at least one read was 99.5%. Quality control statistics are shown in supplementary Table 1.

Raw FASTQ sequencing files were uploaded to TB Profiler version 0.2.1, an online tool to determine drug resistance *in silico*^11^. It uses raw sequence data as input, aligns these to the *M*. *tuberculosis* H37Rv reference genome, and compares identified single nucleotide polymorphisms (SNPs) and indels to a curated list of 1,325 drug resistance mutations. Although it allows examining heteroresistance, we focused on majority variants. In addition, it determines the *M*. *tuberculosis* lineage based on a 62-SNP barcode^12^. The TB Profiler-predicted resistance mutations were validated using PhyResSE, version 1.0^13^, another online tool that maps raw sequencing reads to the *M*. *tuberculosis* H37Rv reference strain to report drug resistance and phylogenetic SNPs. We used the *M*. *tuberculosis* complex numbering system, based on the sequence of the reference strain; H37Rv^14^.

### Phylogeny construction

A phylogeny was constructed to examine clustering of the isolates. The sequence reads were aligned to reference strain *M*. *tuberculosis* H37Rv, accession number NC_000962.3, and variants were called using Breseq software, version 0.27.1^15^. We extracted all 5,980 variable positions across the 72 *M*. *tuberculosis* sequences and concatenated them into a single alignment. Solely for the purpose of creating the phylogenetic tree, SNPs occurring in PE/PPE genes, genes related to mobile elements, as well as genes previously associated with drug resistance^11^ were removed. The remaining 5,794 SNPs were used to construct the phylogenetic tree using PhyML, version 3.0^16^ using the HKY85 model with four categories for the gamma distribution, and using a hundred bootstraps.

### Comparison of MGIT, minimum inhibitory concentrations and whole genome sequencing

For each isolate, susceptibility to STR, INH, RIF, and EMB as determined by MGIT was compared to the MICs determined by MycoTB. If MycoTB MICs were higher than the critical concentration (>0.25 µg/ml for INH, >1.0 µg/ml for RIF, >4.0 µg/ml for EMB, and >2.0 µg/ml for STR^17^, the isolate was considered resistant to the respective drug. Cohen’s kappa statistic was calculated for each drug to determine the level of agreement between MycoTB- and MGIT-determined resistance. In addition, drug resistance mutations identified by TB Profiler and/or PhyResSE were correlated to MGIT results and MICs. We visualized the MIC for each drug in histograms, and subsequently determined the frequencies of the individual resistance-determining mutations and plotted their distributions against the MGIT results for INH, RIF, EMB, and STR, and against the MICs for first- and second-line drugs. For isolates with discrepant susceptibility results for INH or RIF between WGS and MGIT, we compared the MGIT to the DST performed on Löwenstein-Jensen and in case of genotypically resistant, phenotypically susceptible isolates we examined the quality of the variant call (Supplementary Table 2). All analyses were performed in R, version 3.3.1 (http://www.R-project.org).

### Data availability

The raw sequence files (FASTQ) were archived on the NCBI Sequence Read Archive and are available at: (URL will be included after sequence file shave been uploaded). The individual isolates can be accessed under the following Biosample accession numbers: SAMN09402650-SAMN09402721. The Bioproject accession number is: PRJNA475771.

## Results

### Phenotypic drug susceptibility testing

MGIT results for INH, STR, and EMB were available for 68 out of 72 isolates (four cultures being contaminated); RIF MGIT results were available for 69 isolates, with three cultures contaminated. Fifty-seven isolates were phenotypically resistant to INH, 34 to RIF, 24 to STR, and three to EMB. Using the MycoTB microdilution method, MICs for INH, RIF, STR, EMB, ETO, OFL, MXF, PAS, cycloserine, AMK, and KAN were successfully determined for 64 isolates; six samples were contaminated, and two repeatedly failed to grow in the MycoTB plate. By MycoTB, 51 isolates were resistant to INH, 31 to RIF, nine to STR, four to EMB, 40 to ETO, four to OFL, one to MXF, one to PAS, one to cycloserine, two to AMK, and three were resistant to KAN (Supplementary Figure 1). Poly-drug resistance was observed in 47, and MDR-TB in 28 isolates, no isolates were XDR-TB according to MycoTB. The agreement between MGIT and MycoTB for INH, RIF, STR and EMB was greater than to be expected by chance (Supplementary Table 3). Cohen’s kappa indicated excellent agreement for INH (κ = 0.794; p < 0.001) and RIF (κ = 0.905; p < 0.001), and little agreement for STR (κ = 0.289; p = 0.006) and EMB (κ = 0.246; p = 0.045).

### Whole genome sequencing and *in silico* determination of drug resistance

Sixty-three (87.5%) isolates harboured drug resistance-conferring mutations, and 48 (66.7%) had INH and RIF resistance-conferring mutations and were genotypically multidrug-resistant. The identified drug resistance mutations are listed in Table 1. Resistance-conferring mutations to INH were identified in 61 (84.7%) isolates. The most common INH resistance mutation was *katG* S315T, which was present in 38 (52.8%) isolates as a single mutation, and in 16 (22.2%) together with a mutation in the *fabG1* promotor region (synonym: *inhA* promotor region). Resistance-conferring mutations to RIF were found in 50 (69.4%) isolates. The most prevalent RIF resistance-conferring mutation was *rpoB* S450L, observed in 17 (23.6%) isolates as a single mutation, and in one isolate as a combined mutation. Mutations in genes related to EMB susceptibility were identified in 39 (54.2%) isolates; *embB* M306I was observed in 25 (34.7%) isolates, and in 14 of these as a single mutation. Resistance to STR due to mutations in the *rpsL* and *rrs* loci was found in 19 (26.4%) isolates; with *rrs* A1401G in 13 isolates being the most frequent. This mutation however is not considered causative of STR resistance according to expert knowledge^18,19^. Mutations in the *pncA* gene, encoding the target of PZA were found in 23 (31.9%) isolates, 14 of which had a single *pncA* A146V mutation. Mutations to second-line drugs were less frequently observed, the *rrs* A1401G mutation associated with resistance to AMK, CAP, and KAN was most common, together with the C15T mutation in the promotor region of *fabG1* conferring resistance to ETO that was found in 20 (27.8%) isolates. The latter mutation is also associated with INH resistance. Only five isolates had one of four different mutations conferring resistance to FLQs.

**Table 1:** Frequency of *M*. *tuberculosis* resistance-conferring mutations.

Drug	Gene	Mutation	Frequency (%)	MGIT-resistant^#^	MIC > CC^#^
INH	katG	**Ser315Thr**	**38 (52.8)**	**35/35**	**30/30**
	katG + ahpC	Asp311Gly + G-48A promoter	1 (1.4)	1/1	1/1
	katG + fabG1	Ser315 Thr + C-15T promoter	16 (22.2)	15/15	16/16
	fabG1	C-15T promoter	4 (5.6)	4/4	2/4
	fabG1 + inhA	C-15T promoter + Ile21Val	1 (1.4)	1/1	0/1
	ahpC	C-52T promoter	1 (1.4)	1/1	1/1
RIF	rpoB	Asp435Val	4 (5.6)	3/4	3/4
		Gln432Pro	1 (1.4)	1/1	1/1
		His445Asn	12 (16.7)	2/11	1/7
		His445Asp	1 (1.4)	1/1	1/1
		His445Tyr	4 (5.6)	4/4	4/4
		Leu430Pro	2 (2.8)	0/1	0/1
		**Ser450Leu**	**17 (23.6)**	**15/15**	**16/16**
		Ser450Trp	1 (1.4)	1/1	1/1
		Asp435Gly + His445Asn	1 (1.4)	1/1	1/1
		Asp435Val + His445Asn	1 (1.4)	1/1	NA
		His445Asn + Pro454Leu	2 (2.8)	0/2	1/2
		Leu430Pro + His445Asn	1 (1.4)	1/1	NA
		Met434Ile + His445Asn	1 (1.4)	1/1	1/1
		Phe424Leu + His445Asn	1 (1.4)	1/1	0/1
		His445Asn + Ser450Leu + Pro454Leu	1 (1.4)	1/1	0/1
EMB	embA	C-12T promoter	1 (1.4)	0/1	0/1
	embA + embB	C-12T promoter + Asp354Ala	1 (1.4)	0/1	0/1
		C-12T promoter + Glu504Asp	1 (1.4)	0/1	0/1
		C-12T promoter + Met306Ile	1 (1.4)	0/1	0/1
		C-12T promoter + Met306Leu + Asp354Ala	1 (1.4)	0/1	0/1
	embB	Asp354Ala	1 (1.4)	0/1	0/1
		Gly406Asp	5 (6.9)	0/5	0/1
		**Met306Ile**	**14 (19.4)**	**0/12**	**0/14**
		Met306Leu	1 (1.4)	0/1	0/1
		Met306Val	2 (2.8)	0/2	0/1
		Met306Ile + Gly406Asp	9 (12.5)	3/9	4/8
		Met306Val + Asp328Tyr	1 (1.4)	0/1	0/1
		Met306Ile + Tyr319Ser + Asp354Ala	1 (1.4)	0/1	0/1
STR	rpsL	Lys43Arg	1 (1.4)	1/1	1/1
		Lys88Arg	1 (1.4)	1/1	1/1
	rpsL + rrs	Lys43Arg + A1401G	2 (2.8)	2/2	2/2
	rrs	A1401G*	**11 (15.3)**	**6/10**	**1/10**
		A514C	2 (2.8)	2/2	2/2
		C1402T*	1 (1.4)	1/1	0/1
		C517T	1 (1.4)	NA	0/1
PZA	pncA	**Ala146Val**	**14 (19.4)**	NA	NA
		Gln10Arg	1 (1.4)	NA	NA
		Gln10Stop	4 (5.6)	NA	NA
		Gly17Asp	2 (2.8)	NA	NA
		Tyr34Stop	1 (1.4)	NA	NA
		Tyr34Stop + Gly17Asp	1 (1.4)	NA	NA
ETO	ethA	Thr61Met	1 (1.4)	NA	1/1
	fabG1	**C-15T promoter**	**20 (27.8)**	NA	**19/19**
	fabG1 + inhA	C-15T promoter + Ile21Val	1 (1.4)	NA	1/1
FQ	gyrA	Ala90Val	1 (1.4)	NA	NA
		**Asp94Gly**	**2 (2.8)**	NA	1/2 (OFL); 0/2 (MXF)
		Ser91Pro	1 (1.4)	NA	0/1 (OFL); 0/1 (MXF)
	gyrB	Asp461Asn	1 (1.4)	NA	1/1 (OFL); 0/1 (MXF)
AMK	rrs	**A1401G**	**13 (18.1)**	NA	**2/12**
		A514C*	2 (2.8)	NA	0/2
		C1402T	1 (1.4)	NA	0/1
		C517T*	1 (1.4)	NA	0/1
CAP	rrs	**A1401G**	**13 (18.1)**	NA	NA
		C1402T	1 (1.4)	NA	NA
KAN	eis	G-14A promoter	2 (2.8)	NA	1/2
	rrs	**A1401G**	**13 (18.1)**	NA	**1/12**
		C1402T	1 (1.4)	NA	0/1

^*^These mutations possibly have no causative role in conferring drug resistance to the respective drug^18,19^. ^#^Presented as N/N tested, because not all isolates with genotypic DST results had phenotypic DST results. The most frequently found mutations per drug are shown in bold.

### Phylogenetic relatedness of the isolates

A phylogenetic tree was constructed based on 5,794 variable common nucleotide positions among the 72 *M*. *tuberculosis* isolates. Seventy-one isolates belonged to the Euro-American lineage and one belonged to the East-Asian lineage. The phylogeny showed that several isolates were closely related, but did not all harbour the same resistance-conferring mutations (Supplementary Figure 2). Fourteen isolates within the same clade carried the *fabG1* (C-15T promotor), *katG* (S315T), *rpoB* (S450L), *embB* (M306I), and *pncA* (A146V) mutations, but they also harboured different mutations. Another 21 isolates clustered together in the tree and carried the S315T *katG* mutation, twenty of them had the H445N *rpoB* mutation, but apart from that their resistance profile differed. In addition, there were five pairs of neighbouring isolates that had similar resistance mutations.

### Comparison of MGIT, minimum inhibitory concentrations and whole genome sequencing

First, whole genome sequencing was compared with phenotypic DST by MGIT. Supplementary Figure 3 and Table 1 show the comparison of MGIT and WGS resistance prediction. For MGIT, all isolates with well-known INH resistance-conferring mutations were phenotypically resistant to INH, and all isolates without these mutations were susceptible to INH. For RIF, 13/69 (19%) discrepancies were observed between MGIT and WGS; all concerned WGS-resistant, MGIT-susceptible isolates. Eleven of these had a H445N *rpoB* mutation; eleven were susceptible according to DST performed on Löwenstein-Jensen medium, and variant call qualities were good (Supplementary Table 2). For STR, WGS and MGIT did not match for 15/68 (22%) isolates; four had an A1401G *rrs* mutation but were susceptible according to MGIT, and 11 were MGIT-resistant, but had no STR resistance-conferring mutations. For EMB, we observed 34/69 (49%) discrepancies; all isolates without an EMB resistance-conferring mutation were susceptible, but 34 isolates with an EMB resistance mutation were also susceptible. Only isolates with the combination of two *embB* mutations (M306I and G406D) were MGIT EMB-resistant, and this was only the case for 3 out of 9 isolates with this combined mutation.

**Figure 1:**
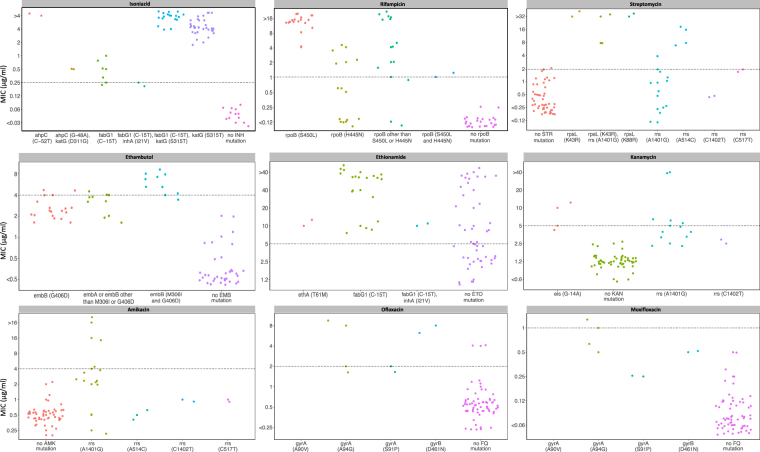
Drug resistance mutations with corresponding minimum inhibitory concentrations for nine anti-tuberculous drugs. Each dot represents an isolate and is coloured by mutation. *AhpC* C-52T, G-48A, *fabG1* C-15T, and *eis* G-14A are mutations in the promotor area of the respective gene. The y-axes show the minimum inhibitory concentrations in μg/ml. NOTE. Dots may be shown in-between tested MICs to increase readability.

We next compared WGS with the MycoTB assay, which yields MICs (Fig. 1 and Table 1). Different resistance mutations were associated with different MIC ranges. For INH, the *katG* S315T mutation was associated with clearly elevated MICs, as was the *ahpC* C52T promotor mutation although it only occurred in one isolate. Other mutations in *ahpC*, *fabG1*, and *inhA* were associated with MICs around or just above the critical concentration. For RIF, the *rpoB* S450L mutation was associated with elevated MICs, but other *rpoB* mutations were associated with a range of MICs including the critical concentration. Mutations in *rpsL* were all associated with MICs above the critical concentration for STR and the same applies to the *rrs* A514C mutation. However, other *rrs* mutations were associated with MICs around or below the critical concentration for STR. All found mutations in *embA* or *embB* were associated with elevated EMB MICs; their ranges included the critical concentration. All isolates with resistance-conferring mutations to ETO had elevated MICs. The *fabG1* C15T promotor mutation was associated with a bimodal MIC distribution for ETO; some isolates had an MIC of twice the critical concentration; most others with the mutation had an eight times higher MIC than the critical concentration. For AMK and KAN, all observed mutations were associated with an MIC range below or including the critical concentration. OFL or MXF resistance mutations were uncommon, and these mutations were associated with an MIC above the critical concentration in only two isolates, and only for OFL. For STR and OFL, but especially for ETO, we observed isolates with no mutations conferring resistance to the respective drug, but with MICs above the critical concentration.

## Discussion

In this study, we demonstrate that MIC determination using SensiTitre MycoTB is an excellent alternative to conventional DST methods; it is relatively rapid, straightforward, and it provides quantitative data on susceptibility to first- and second-line drugs, thus facilitating therapeutic decision-making and therapeutic drug monitoring to optimize regimen efficacy and minimize toxicity^20^. Whole genome sequencing has been shown to be a sensitive, accurate, rapid, and financially feasible method for *M*. *tuberculosis* drug susceptibility testing^21^. Here we show that WGS has additional added value in terms of predicting the level of resistance to different drugs. For key anti-tuberculosis drugs including rifampicin, isoniazid, ethambutol and fluoroquinolones, distinct genomic mutations are associated with particular MIC ranges, some including the critical concentration.

The MycoTB microdilution method is a commercially available method for MIC testing in *M*. *tuberculosis* and the first for second-line drugs. Previous studies have tested its performance compared to currently available methods for drug susceptibility testing, such as the BACTEC MGIT 960^22^, the indirect agar proportion method^8,9,23^, the Löwenstein-Jensen proportion method^24^, genotypic tests^25^, or a combination of these^17^, and it has proven a rapid, convenient, quantitative, and accurate method for testing both first- and second-line antituberculosis drug susceptibility. Although we found discrepancies with WHO-endorsed phenotypic tests for first line drugs, especially STR and EMB, phenotypic DST has been proven to be difficult for these drugs and the value of these tests as ‘gold standard’ is questionable. Compared to previous studies comparing MycoTB with MGIT, the MycoTB in the present study performed slightly better for INH and RIF, comparable for EMB, and slightly worse for STR^17,22^. Furthermore, the microwell plate format without the need for equipment will allow its use in resource-pour settings, where it is most needed, provided that culture-based DST is possible, and proper biosafety measures are taken. Moreover, the visual reading of the plates requires some operator experience.

Whole genome sequencing is revolutionizing drug susceptibility testing in tuberculosis and has great potential in public health interventions. However, the various platforms used, as well as the shortcomings of critical concentrations for phenotypic DST hinder the comparison of genotypic and phenotypic DST, hence the validation of WGS-based susceptibility testing^26^. The current critical concentrations have a limited evidence base and stem largely from observations on wild type MIC distributions, not from clinical or pharmacokinetics/pharmacodynamics studies^27^. Data from several studies indicate that these critical concentrations need to be revised because they either bisect the wild-type distribution, or are substantially higher than the MICs of wild-type organisms resulting in potential false reporting of susceptibility^28^. Indeed, in the current study, we observed that isolates with mutations associated with decreased susceptibility to RIF, STR, and EMB were reported susceptible according to MGIT, and MICs for these drugs were often around the critical concentration. These data support the notion of the European Committee on Antimicrobial Susceptibility Testing (EUCAST) that critical concentrations should be defined by combining MIC distributions, preferably combined with clinical outcomes and pharmacokinetics/pharmacodynamics data^28,29^. Even though WGS has already been successfully implemented in routine diagnostic practice in some settings and has been shown to achieve generally high agreement with phenotypic first-line DST^21,30,31^, MIC testing may help in more accurately assessing the performance of WGS for drug resistance detection and the role of this method in TB laboratory diagnosis.

The discrepancies between MGIT and MICs observed in our study support the current dogma that inconsistencies between phenotypic and genotypic DST found in important studies investigating the use of WGS for predicting *Mycobacterium tuberculosis* drug susceptibility are partly attributed to shortcomings with currently endorsed methods of phenotypic DST, which essentially provide only qualitative results (sensitive or resistant), especially where mutations conferring low-level drug resistance are involved^32^. However, it is important to appreciate that unknown resistance mechanisms, inadequate limits of detection or artefacts of sequencing, random errors, and false associations between genotype and phenotype due to epistatic interactions could all play a role^33–35^. The clinical impact of these discrepancies, and the effect of treatment regimen based on different DST methods are not clear yet, and clinical validation of the influence of MICs of single drugs on treatment outcome is warranted. The analysis of *embB* mutations has long been considered futile because they did not match well with phenotypic DST results^36^, our data however show that these mutations do have an effect on MICs thus potentially influence treatment efficacy^37,38^ as has been shown previously in a mouse model of aerogenic tuberculosis^39^.

Similar to what has been shown in a recent study by Heyckendorf *et al*.^32^, we observed that WGS did not miss phenotypically confirmed resistances to first-line drugs, except for STR. We discovered that the MIC-range for isolates without STR resistance-conferring mutations was wide and included the critical concentration. Unknown mutations in these isolates could have caused resistance. Critical concentration artefacts could also explain the discrepancies; 16 isolates were MGIT-resistant for STR, but had an MIC below the critical concentration. In addition, WGS predicted resistance in a number of phenotypically susceptible isolates. Especially *rpoB* H445N, *embB* G406D and M306I, as well as the *rrs* mutations did not correlate well with phenotypic resistance, in line with previous findings^19,26,40–42^. We found that these mutations increased the MIC only slightly, or were associated with wide MIC-ranges. However, previous studies have shown that these mutations may still be clinically relevant^37,39,41,43,44^.

This is the first study to investigate drug resistance in *M*. *tuberculosis* isolates from the greater Craiova area, Romania. In this selection of phenotypically drug-resistant isolates we observed low frequencies of resistance to second-line drugs, possibly as a result of limited availability of second-line anti-TB drugs^7^. Surprising was the observation that all-but-one of the isolates belonged to the Euro-American lineage, because there has been an extremely large cluster of MDR/XDR-TB *Mycobacterium tuberculosis* strains in the EU, especially in the eastern part, which is significantly related to the spread of one strain or clone of the Beijing genotype^45^.

The relatively small number of isolates does not permit drawing solid conclusions on the effect of most drug resistance mutations on drug susceptibility and the interrelatedness of different mutations, or their combined effects on the level of resistance. However, the findings we present are an important contribution to the field because of a lack of data correlating *M*. *tuberculosis* genotype and MICs^46^. In addition, the phylogeny showed one clade of genetically closely related isolates. This could have affected the MIC distribution of the drugs these isolates are resistant to. However, the drug resistance mutations found in these isolates differed to an extent that makes it unlikely that these isolates represent the same strain. Also, we could not associate drug resistance mutations with the corresponding MICs for two strains that failed to grow on the MycoTB microtiter plate and six that were contaminated. Lastly, pyrazinamide is not included in the MycoTB plate, and we could not assess the MICs related to pyrazinamide resistance-associated mutations, which were discovered in 23 (32%) isolates.

In summary, we have shown that, especially for RIF, STR, and EMB, MICs near the critical concentration are common. Consequently, phenotypic DST based on critical concentration testing, e.g. the MGIT method, may provide inaccurate results, possibly leading to suboptimal treatment regimens. We compared WGS-predicted drug resistance mutations directly with MICs and found that different mutations lead to different levels of resistance; knowing the underlying mutations can guide clinical decision-making and facilitate therapeutic drug monitoring, ultimately leading to better treatment outcome.

## Electronic supplementary material

Supplementary figures and tables
