# Supplementary material for: Linking minimum inhibitory concentrations to whole genome sequence-predicted drug resistance in Mycobacterium tuberculosis strains from Romania
Source: Sci Rep. 2018 Jun 26;8:9676. doi: 10.1038/s41598-018-27962-5 (PMC6018741; doi:10.1038/s41598-018-27962-5)
Supplement: Supplementary file 1 — Supplementary figures and tables [file 41598_2018_27962_MOESM1_ESM.docx]

**Linking minimum inhibitory concentrations to whole genome sequence-predicted drug resistance in *Mycobacterium* *tuberculosis* strains from Romania**

**Authors**

Carolien Ruesen, Anca Lelia Riza, Adriana Florescu, Lidya Chaidir, Cornelia Editoiu, Nicole Aalders, Dragos Nicolosu, Victor Grecu, Mihai Ioana, Reinout van Crevel, Jakko van Ingen

***Figure Legends***

**Supplementary Figure 1.** **Overview of minimum inhibitory concentrations for nine anti-tuberculous drugs.** Minimum inhibitory concentrations were determined using the MycoTB microdilution method for 64 *M. tuberculosis* isolates.

**Supplementary Figure 2. Phylogenetic tree showing the genetic relatedness of 72 *M. tuberculosis* strains isolated from 72 Romanian patients.**

**Supplementary Figure 3.** **Comparison of whole genome sequencing and Mycobacterium Growth Indicator Tube to determine drug susceptibility to first-line anti-tuberculous drugs.**

**Supplementary Figure 1.** Overview of minimum inhibitory concentrations for nine anti-tuberculous drugs.

**Supplementary Figure 2.** Phylogenetic tree showing the genetic relatedness of 72 *M. tuberculosis* strains isolated from 72 Romanian patients.

Euro-American

East-Asian

**Supplementary Figure 3.** Comparison of whole genome sequencing and Mycobacterium Growth Indicator Tube to determine drug susceptibility to first-line anti-tuberculous drugs.

Number of isolates

**Supplementary Table 1.** Description of sequencing quality control parameters and statistics.

| **Study number** | **Clean reads** | **Q20%** | **Total bases** | **Mean coverage** | **Median coverage** | **Percentage bases >1** |
| --- | --- | --- | --- | --- | --- | --- |
| MTB0083 | 5010168 | 98,8 | 729245204 | 155,8 | 160 | 99,6 |
| MTB0085 | 9244564 | 98,9 | 1331280683 | 292,3 | 299 | 99,4 |
| MTB0153 | 4980076 | 98,8 | 720076577 | 157,9 | 161 | 99,5 |
| MTB0159 | 4326888 | 98,8 | 628038812 | 138,1 | 142 | 99,6 |
| MTB0167 | 5439392 | 98,9 | 792319455 | 174,2 | 178 | 99,6 |
| MTB0210 | 5880320 | 98,9 | 852347773 | 187,4 | 192 | 99,5 |
| MTB0269 | 4613254 | 98,8 | 671851858 | 147,7 | 151 | 99,7 |
| MTB0326 | 5072238 | 98,8 | 730714611 | 160,2 | 164 | 99,5 |
| MTB0373 | 4397414 | 98,8 | 639876834 | 139,8 | 143 | 99,5 |
| MTB0397 | 2668762 | 98,8 | 388958930 | 82,6 | 85 | 99,8 |
| MTB0398 | 3282760 | 98,7 | 479249813 | 103,1 | 105 | 99,5 |
| MTB0421 | 5498680 | 98,8 | 797711420 | 174,6 | 179 | 99,5 |
| MTB0455 | 2581286 | 98,8 | 377936416 | 79,3 | 78 | 99,5 |
| MTB0472 | 6458404 | 98,8 | 937346421 | 204,5 | 210 | 99,5 |
| MTB0477 | 5466848 | 98,8 | 793482681 | 173,6 | 178 | 99,5 |
| MTB0498 | 3500310 | 98,7 | 509420826 | 108,6 | 112 | 99,5 |
| MTB0565 | 6226184 | 98,8 | 907091707 | 198,6 | 204 | 99,5 |
| MTB0598 | 5334456 | 98,7 | 776163802 | 169,7 | 173 | 99,3 |
| MTB0621 | 1315430 | 98,7 | 192150033 | 40,4 | 41 | 99,4 |
| MTB0670 | 1723318 | 98,7 | 252717866 | 51,7 | 53 | 99,5 |
| MTB0679 | 5047768 | 98,8 | 735251932 | 160,7 | 164 | 99,7 |
| MTB0681 | 2592134 | 98,3 | 374789416 | 81,0 | 82 | 99,5 |
| MTB0690 | 2788926 | 98,7 | 407670628 | 88,0 | 90 | 99,5 |
| MTB0704 | 4512874 | 98,8 | 655016172 | 143,0 | 146 | 99,4 |
| MTB0717 | 5180780 | 98,8 | 753070029 | 164,8 | 169 | 99,5 |
| MTB0731 | 6910880 | 98,8 | 995135438 | 218,9 | 225 | 99,7 |
| MTB0750 | 16678804 | 98,8 | 2289895133 | 501,3 | 500 | 99,6 |
| MTB0751 | 5024722 | 98,8 | 725893624 | 159,6 | 160 | 99,6 |
| MTB0777 | 6829294 | 98,8 | 984893721 | 216,8 | 222 | 99,6 |
| MTB0805 | 6349612 | 98,9 | 920541812 | 202,5 | 208 | 99,8 |
| MTB0808 | 13982680 | 98,9 | 1992277055 | 438,3 | 450 | 99,6 |
| MTB0819 | 7285102 | 98,8 | 1045110908 | 228,2 | 233 | 99,3 |
| MTB0820 | 6437616 | 98,8 | 928788036 | 203,9 | 209 | 99,6 |
| MTB0822 | 10553814 | 98,8 | 1498369699 | 329,4 | 335 | 99,6 |
| MTB0835 | 13273726 | 98,9 | 1867111990 | 410,9 | 422 | 99,5 |
| MTB0838 | 11449646 | 98,9 | 1631527546 | 358,9 | 368 | 99,6 |
| MTB0844 | 7042508 | 98,8 | 1014627854 | 223,5 | 229 | 99,6 |
| MTB0869 | 9514364 | 98,8 | 1348716932 | 296,8 | 304 | 99,6 |
| MTB0890 | 9986614 | 98,8 | 1426980119 | 312,9 | 321 | 99,5 |
| MTB0898 | 2673806 | 98,8 | 389093509 | 78,8 | 80 | 99,5 |
| MTB0899 | 3748466 | 98,8 | 543659909 | 110,8 | 111 | 99,6 |
| MTB0902 | 5105332 | 98,8 | 738012399 | 161,6 | 166 | 99,5 |
| MTB0935 | 4166006 | 98,8 | 600850392 | 131,6 | 134 | 99,9 |
| MTB0940 | 6345710 | 98,9 | 912105253 | 200,3 | 205 | 99,8 |
| MTB0954 | 5182548 | 98,9 | 749833435 | 164,1 | 169 | 99,3 |
| MTB0955 | 6342280 | 98,9 | 912264427 | 200,4 | 206 | 99,4 |
| **Study number** | **Clean reads** | **Q20%** | **Total bases** | **Mean coverage** | **Median coverage** | **Percentage bases >1** |
| MTB0968 | 5597674 | 98,8 | 808331240 | 177,3 | 182 | 99,6 |
| MTB0971 | 4810290 | 98,8 | 697511432 | 153,1 | 158 | 99,5 |
| MTB0998 | 6125292 | 98,8 | 882233413 | 193,8 | 200 | 99,5 |
| MTB1063 | 3131132 | 98,8 | 452357038 | 99,1 | 102 | 99,5 |
| MTB1085 | 4055568 | 98,8 | 586012462 | 127,2 | 131 | 99,5 |
| MTB1091 | 4496052 | 98,8 | 651171048 | 143,0 | 147 | 99,5 |
| MTB1094 | 5037192 | 98,8 | 726608727 | 159,5 | 164 | 99,5 |
| MTB1105 | 2766362 | 98,8 | 403334631 | 86,0 | 88 | 99,5 |
| MTB1114 | 7021708 | 98,8 | 1005915329 | 221,2 | 227 | 99,7 |
| MTB1119 | 4133620 | 98,7 | 598668119 | 131,1 | 134 | 99,5 |
| MTB1122 | 5510106 | 98,8 | 800687586 | 175,7 | 179 | 99,5 |
| MTB1132 | 2041418 | 98,7 | 297936222 | 64,9 | 66 | 99,2 |
| MTB1134 | 2636480 | 98,8 | 384682814 | 84,1 | 86 | 99,3 |
| MTB1153 | 2592910 | 98,8 | 377531940 | 82,8 | 85 | 99,5 |
| MTB1181 | 3687154 | 98,8 | 536107686 | 117,5 | 121 | 99,5 |
| MTB1183 | 4231256 | 98,8 | 614868223 | 135,1 | 138 | 99,5 |
| MTB1184 | 1520090 | 98,8 | 222159082 | 47,7 | 49 | 99,5 |
| MTB1186 | 2331846 | 98,7 | 339340987 | 74,3 | 76 | 99,5 |
| MTB1195 | 5434990 | 98,7 | 786889583 | 172,7 | 177 | 99,6 |
| MTB1201 | 3044342 | 98,7 | 439656581 | 96,5 | 99 | 99,5 |
| MTB1209 | 3420894 | 98,7 | 494659829 | 108,5 | 111 | 99,5 |
| MTB1221 | 2829856 | 98,8 | 411306820 | 89,8 | 91 | 99,5 |
| MTB1364 | 3977738 | 98,7 | 576100368 | 125,7 | 128 | 99,5 |
| MTB1466 | 2388624 | 98,7 | 347922516 | 75,6 | 77 | 99,6 |
| MTB1509 | 3914664 | 98,6 | 568644763 | 124,2 | 126 | 99,5 |
| MTB1523 | 2993102 | 98,8 | 435834604 | 95,7 | 98 | 99,5 |

**Supplementary Table 2.** Discrepant drug susceptibility test results according to whole genome sequencing and Mycobacterium Growth Indicator Tube

| Isolate | Discrepant WGS result | Discrepant MGIT result | LJ result | **Sequencing statistics** | | |
| --- | --- | --- | --- | --- | --- | --- |
|  |  |  |  | Underlying mutation | Sequencing coverage depth (no. of reads) | Percentage of reads supporting the variant base |
| MTB0159 | RIF-resistant | RIF-susceptible | RIF-susceptible | *rpoB* (H445N) | 147 | 100% |
| MTB0498 | RIF-resistant | RIF-susceptible | RIF-susceptible | *rpoB* (H445N) | 115 | 96.5% |
| MTB0598 | RIF-resistant | RIF-susceptible | Rif-resistant | *rpoB* (D435V) | 161 | 97.5% |
| MTB0621 | RIF-resistant | RIF-susceptible | Rif-resistant | *rpoB* (H445N)  *rpoB* (P454L) | 37  48 | 100%  100% |
| MTB0681 | RIF-resistant | RIF-susceptible | RIF-susceptible | *rpoB* (H445N) | 123 | 97.6% |
| MTB0819 | RIF-resistant | RIF-susceptible | RIF-susceptible | *rpoB* (L430P) | 234 | 99.6% |
| MTB0838 | RIF-resistant | RIF-susceptible | RIF-susceptible | *rpoB* (H445N) | 383 | 98.4% |
| MTB0898 | RIF-resistant | RIF-susceptible | RIF-susceptible | *rpoB* (H445N) | 78 | 97.4% |
| MTB0899 | RIF-resistant | RIF-susceptible | RIF-susceptible | *rpoB* (H445N) | 122 | 99.2% |
| MTB1085 | RIF-resistant | RIF-susceptible | RIF-susceptible | *rpoB* (H445N) | 130 | 99.2% |
| MTB1091 | RIF-resistant | RIF-susceptible | RIF-susceptible | *rpoB* (H445N) | 136 | 99.3% |
| MTB1153 | RIF-resistant | RIF-susceptible | RIF-susceptible | *rpoB* (H445N) | 79 | 100% |
| MTB1195 | RIF-resistant | RIF-susceptible | RIF-susceptible | *rpoB* (H445N) | 183 | 99.5% |

**Supplementary Table 3.** Concordance between Mycobacterium Growth Indicator Tube and MycoTB.

|  | | | MycoTB | |  |  |
| --- | --- | --- | --- | --- | --- | --- |
|  |  |  | MIC above breakpoint (resistant) | MIC below breakpoint (susceptible) | % Categorical agreement | Cohen’s kappa (p-value) |
| MGIT | INH | Resistant | 48 | 3 | 93.5 | 0.794 (p<0.001) |
|  |  | Susceptible | 1 | 10 |  |  |
|  | RIF | Resistant | 29 | 2 | 95.2 | 0.905 (p<0.001) |
|  |  | Susceptible | 1 | 31 |  |  |
|  | STR | Resistant | 7 | 16 | 71.0 | 0.289 (p=0.006) |
|  |  | Susceptible | 2 | 37 |  |  |
|  | EMB | Resistant | 1 | 2 | 92.3 | 0.246 (p=0.045) |
|  |  | Susceptible | 3 | 59 |  |  |
